# Influence of the Stainless-Steel Microstructure on Tribological Behavior and Surface Integrity after Ball Burnishing

**DOI:** 10.3390/ma15248829

**Published:** 2022-12-10

**Authors:** Alejandra Torres, Nuria Cuadrado, Jordi Llumà, Montserrat Vilaseca, J. Antonio Travieso-Rodriguez

**Affiliations:** 1 Eurecat, Centre Tecnològic de Catalunya, Unit of Metallic and Ceramic Materials, Plaça de la Ciència 2, 08243 Manresa, Spain; 2 Department of Mechanical Engineering, Universitat Politècnica de Catalunya, Av. Eduard Maristany 10-14, 08019 Barcelona, Spain; 3 Department of Science and Material Engineering, Universitat Politècnica de Catalunya, Av. Eduard Maristany 10-14, 08019 Barcelona, Spain

**Keywords:** ball burnishing, tribological interaction, stainless steel, friction coefficient, surface integrity

## Abstract

Burnishing is a plastic deformation process that reduces roughness while increasing hardness by introducing compressive residual stresses near the surface zone. These improvements will depend mainly on two fundamental variables: the applied load and the friction derived from the tool–surface interaction. Nevertheless, microstructural differences in the materials have not yet been considered within this interaction. This leads to a generalization of the process that can result in the failure of industrial components. Therefore, the aim of this work is to study the microstructural influence of the ball-burnishing process from a tribological perspective. Thus, martensitic and austenitic stainless steels were evaluated in terms of friction and surface integrity. The results show that parameterizing the process according to the tool–surface interaction is critical since improvements depend on friction as a function of the availability of plastic deformation of the crystallographic structures.

## 1. Introduction

Several industries make extensive use of steel components, among which are two different microstructure designations that are distinctive: austenitic AISI 316 and martensitic UNS S46500 stainless steels [1,2]. Since turning and milling are the conventional processes to machine these materials, the presence of irregularities and defects is inherent. This unevenness on metal surfaces causes considerable energy dissipation (friction), surface damage, and fracture during the service life of these components [3]. To minimize these issues, a reduction in roughness and an increase in mechanical properties are required [4,5]. Accordingly, numerous final machining operations have been proposed as applicable solutions, among them, the ball-burnishing process [6,7].

This procedure confers extra mechanical properties on the treated pieces, maintaining low costs and reducing execution times [7,8,9,10]. Properties such as strain hardening are amplified on a metal surface due to the plastic deformation prompted by the displacement of an indenter at a given pressure [7,10]. At the same time, wear, corrosion, and fatigue resistance are improved due to the newly induced residual compressive state [7,8]. Furthermore, the surface appearance is enhanced because of the decrease in roughness [6,10,11]. However, to achieve these advantages, process (overloading) and material limitations (loss of ductility) [12], which depend on the microstructure [13,14,15], must be overcome by using a satisfactory configuration [10,11,12,13,16,17,18]. Industrial components, such as lasting valve seals, pistons, bearing bores, and shafts for pumps, are burnished to reduce friction and noise levels and increase their service life [17,18]. Nevertheless, the burnishing of reinforced martensitic stainless steel (such as UNS S46500) components has not been studied so far, enabling innovation in the improvement of this material’s surfaces. UNS S46500 is a stainless steel characterized by the presence of Ni_3_Ti nanometric precipitates in a martensitic matrix [2]. The few previous works quote the process’s ability to introduce a deep, highly compressive layer into steel surfaces of this nature (martensitic), with a maximum value location (subsurface level) that is mainly influenced by the applied load and, to a lesser extent, by the speed, feed rate, and number of passes [13]. High loads result in shear stress toward the surface [12]. However, an undue load leads to excessive shear stress and therefore premature degradation of the surface finish [11,12,16,17,18]. Thus, experimental approaches and simplified predictable models have been conceptualized in order to set the parameters for the process in the search for good surface quality (smooth finish) and an optimum surface residual stress state. For instance, a prior study established a correlation between the roughness and the compressive layers. The interrelation between martensitic wear volume and residual stresses showed a strong inversely proportional linear dependence [19]. In most cases, an inverse relationship between the skewness parameter (Ssk) and wear volume is also recognized [19]. On the other hand, models have achieved a reliable roughness prediction, but only allow for qualitative adjustment (inaccurate results) in terms of residual stresses [20].

These hits and misses address the study of the burnishing-induced plastic deformation phenomenon as a tribological interaction in which it is essential to consider the first basic integral parameter that governs the process: friction. It is the tribo-contact between the burnishing ball and the machined material (roughness, microstructure, and mechanical properties) that determines the intensity of the strain-induced behavior at the material subsurface [12]. The high friction generated by an increase in the load leads to an induced stress state in the leading bulge similar to that induced by uniaxial compression loading. In contrast, the rear zone behind the indenter reacts as if a uniaxial tension is imposed. The higher the friction coefficient, the shallower the maximum shear stress at the sub-surface. Consequently, the plastic strain is concentrated in a thinner surface layer. Nevertheless, overextended friction values could lead to surface decline (fracture, tensile residual stresses) [12]. When friction decreases, the depth of the maximum shear strain increases (reducing the efficiency of cold-work nanostructuring) [12] and could eventually promote residual stress relaxation, reducing crack propagation inhibition [21]. Thus, the burnishing tribo-interaction defines the geometry (by the plastic deformation degree) and the maximum residual tensor location (by the shear-stress depth). In this regard, the tribological interaction between the ball and the rough surface during the ball-burnishing process is tackled numerically through simulations. Amini et al. [21] developed a model that takes into account the alterations of the friction coefficient between the ball and an extruded ferritic AISI 1038 steel surface. Depending on the defined preload, a low friction coefficient could not spawn significant advancements in the roughness and compression stress state. By contrast, a high friction coefficient could lead to an intensification in the pile-up and a decline in the stress state. This means that a factual friction coefficient must feed the models in order to reproduce and enhance the final surface integrity required for industrial components [19,21]. Moreover, Amini et al. proved that the direction of the highest induced residual stress concentration depends on the burnishing route, regardless of the initial stress state produced by machining [21]. The utmost burnishing effect is made in the perpendicular direction to the process, which means that the burnishing process can induce anisotropic properties in the target piece, in agreement with its final application [19,22]. Consequently, each parameter needs to be established according to the use of the piece, prioritizing the geometric (roughness) or metallurgic (hardness and compression stress state) characteristics [13,22,23]. Therefore, both the micro- and macro-responses to the process must be investigated through the microstructure’s influence on friction behavior.

Consequently, this study reveals that surface improvements (finish and residual stress state) also depend on the tribological interaction degree between the ball and a defined microstructure. Thus, this tribological interaction is now conceptualized numerically by the friction coefficient. Accordingly, a reinforced martensitic stainless steel matrix and an austenitic stainless steel textured surface are evaluated under the same milling and burnishing process conditions (in agreement with the machining conditions applied to the already characterized ferritic AISI 1038 steel [11,21,23,24,25]) in terms of friction and surface integrity using a scratch test procedure, 3D optical profilometry (surface finish), and X-ray diffraction (XRD) technique (residual stresses induced by cold working). The results show that under the same machining configurations, the induced surface integrity depends on the self-hardening coefficient due to the different tribo-contacts during the execution of the burnishing while providing reliable inputs for future integral modeling and process parameterization. Therefore, the interaction of the ball with an established macro-texture is not enough to generalize the process; it is necessary to consider the contact at the micrometric level to define the burnishing applicability.

## 2. Materials and Methods

### 2.1. Materials

Austenitic AISI 316 stainless steel (processed according to EN 10028-7-2016) and martensitic precipitation-hardened UNS S46500 stainless steel (aged according to ASTM A564/A564M) were selected for the present study.

#### 2.1.1. Chemical Composition and Material Processing

Table 1 shows the chemical composition of the analyzed materials using spark emission spectrometry (SPECTROMAXx LMF08, SPECTRO, Kleve, Germany).

#### 2.1.2. Microstructural Characterization

**Figure 1:**
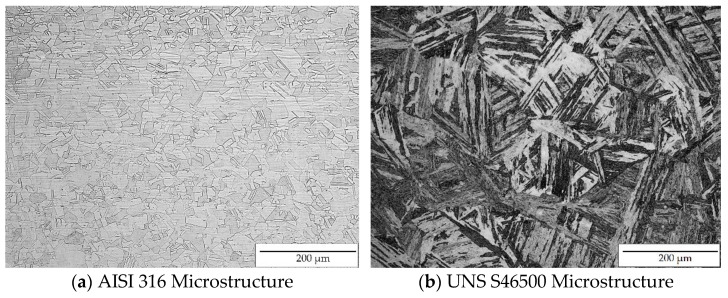
Stainless steel microstructural characterization.

After mechanical polishing to a mirror-surface finish (0.03 µm colloidal silica suspension), the AISI 316 and UNS S46500 samples were etched with aqua regia solution and Kalling I reagent, respectively. Microstructural characterization was performed using optical microscopy (Epiphot 200, Nikon, Tokyo, Japan) (Figure 1). The AISI 316 microstructure consisted of austenitic grains, some of which exhibited twinning, whereas the UNS S46500 stainless steel showed mainly a martensitic matrix.

#### 2.1.3. Hardness

The hardness values of the austenitic AISI 316 and martensitic UNS S46500 stainless steels were 168 ± 6 HV and 521 ± 10 HV, respectively. The values were the mean of 10 measurements acquired using the Vickers microindentation test at a load of 1000 g (Micrometer HV1, Future Tech, FM-700, Tokyo, Japan). Ferritic AISI 1038 steel, used as a reference in this study, has a hardness value of 175 HV ± 10 [21].

#### 2.1.4. Surface Roughness

In the first step, the specimens were subjected to the milling conditions indicated in Table 2 using a CNC router milling machine (LAGUN 600, MAHER HOLDING, Legutiano, Spain). The macro-texture surface parameters after the milling and burnishing processes were acquired using an Alicona microscope (InfiniteFocusSL, Bruker, Karlsruhe, Germany) and further processed with image analysis software (Mountains 5.1.1.5944, Digital Surf, Besançon, France) according to the ISO-25178-2:2016 standard [26].

In order to assess the surface changes due to the burnishing process, the 3D roughness parameters were computed for each of the studied materials. Accordingly, the arithmetical mean height (Sa), root mean square height (Sq), skewness (Ssk), kurtosis (Sku), texture aspect ratio (Str), and ten-point height (S10z) were processed.

### 2.2. Experimental Methods

#### 2.2.1. Ball-Burnishing Process

**Figure 2:**
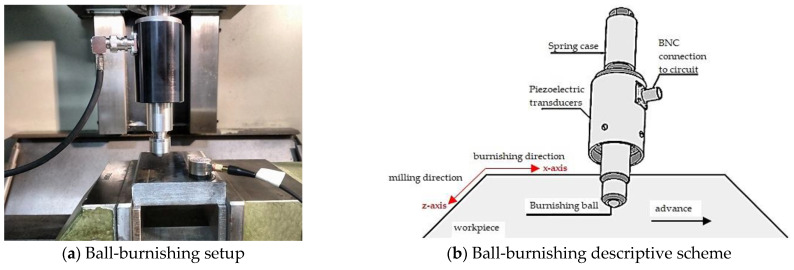
Ball-burnishing configuration.

Previous works on ferritic milled surfaces provided the path for the ball-burnishing configuration of the austenitic and martensitic microstructures [11]. Nevertheless, due to the low hardening coefficient and high hardness conferred by the martensitic matrix, UNS S46500 required a load increase in contrast to AISI 316. The ball-burnishing process was performed using a hard, metal ball with a 10 mm diameter adapted to the force transmission unit of the *Acoustomill* tool (Spanish patent number 201730385) [27]. The process setup and its descriptive scheme are shown in Figure 2.

The set assembled in the CNC router milling machine was displaced once in the perpendicular direction (x-axis) to the milling finish (z-axis) on a 10 mm × 10 mm patch for each material. The ball-burnishing operational parameters are summarized in Table 3.

#### 2.2.2. Uniaxial Tensile Properties

The elastic properties were established using an ultrasonic method (Panametrics 5900 PR pulser, Olympus, Tokyo, Japan) and an oscilloscope (Hameg HM1508, RS, Corby, UK). The longitudinal plastic tensile properties were acquired using the conventional tensile test configuration (ISO 6892-1 standard) [28]. Three AISI 316 and five UNS S46500 tensile specimens fitted to the standard requirements (width = 6 mm and Lc = 34 mm) [28] were tested. The strain measurements (0.0067 s^−1^ until the failure) were obtained by a video-extensometer device. Table 4 shows the measured mechanical properties for both materials.

#### 2.2.3. Friction Coefficient

The interaction of the tool and the textured surface was resolved according to the classical Hertzian theory of non-adhesive contact [29]. The pressure in the center of the contact region (p_0_) was computed as a function of the normal force (F), the indenter radius (R), and the reduced elastic modulus (E*), in agreement with Equation (1), and applied to the normal contact between a rigid sphere and an elastic half-space.

*p*_0_^3^ = 6F∙E*^2^/π^3^∙R^2^ (1)

E* was defined using Young’s modulus (E_1_, E_2_) and the Poisson coefficient (ν_1_, ν_2_) of the interacting materials according to Expression (2):

1/E* = [(1 − ν_1_^2^)/E_1_] + [(1 − ν_2_^2^)/E_2_] (2)

**Figure 3:**
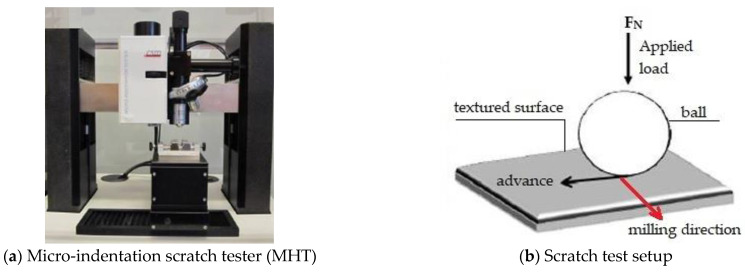
Scratch test configuration.

The pressure values under the cited conditions in 2.2.1 were 2700 MPa for AISI 316 and 2700 MPa and 3100 MPa for UNS S46500. Friction test configurations were adopted for the sequence for the design of laboratory friction and wear proposed by the American Society of Materials (ASM) [30]. The coefficient of friction (COF) resulting from the interaction between the indenter ball and the milled surfaces of the studied steels was measured using a scratch test (Micro-Indentation Scratch Tester (MHT), CSM Instruments, Filderstadt, Germany). In order to achieve the same pressures at the laboratory scale, three linear scratches of 20 mm in length at 600 mm/min by a Tungsten carbide ball indenter of a 2.5 mm diameter were performed under dry conditions. The micro-indentation scratch tester (MHT) and the scratch test’s descriptive scheme are shown in Figure 3.

#### 2.2.4. Surface Integrity Characterization

##### Residual Stresses

Residual stress components (σx, σz) up to a 4 μm depth were obtained using X-ray diffraction equipment (PANalytical—model X’Pert-PRO-MRD, UCDavis, Davis, CA, USA) according to the sin²Ψ mode Ω-tilt method. The point detector (pixel size of 255 µm × 255 µm) was assembled on a parallel plate collimator with a 0.27° angular opening and a planar graphite secondary monochromator. It is well known that machining and finishing operations can induce a phase transformation in austenitic steels. Then, the X-characterization of the milled and burnished austenitic and martensitic surfaces was performed in the reflection (211) of the bcc phase (martensite). The fcc phase corresponding to austenitic steels was not found up to a 4 μm depth. This indicates that martensitic transformation occurs during the milling process. Therefore, the final conditions on the austenitic surface are not influenced by a phase change.

## 3. Results

### 3.1. Friction

**Figure 4:**
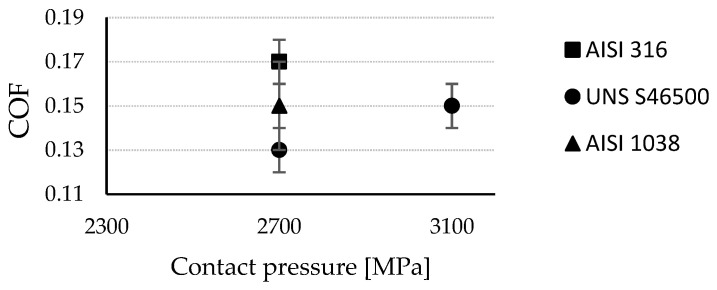
COF at different contact pressures.

In order to evaluate the microstructural response to the tribo-contact during the burnishing process, the COF was computed for each of the studied stainless steels. Figure 4 summarizes the COFs generated by the interactions of austenitic and martensitic milled textured surfaces as a function of the contact pressure. The friction on the already characterized AISI 1038 ferritic steel textured surface [11] was established for comparison purposes.

Under the same ball-burnishing configuration, the steels’ microstructure responses were not coincident. The COF on austenitic steel (0.17) exceeded the COF on ferritic steel (0.15) by 15%. The gap between the austenitic (0.17) and martensitic (0.13) stainless steels’ COFs amounted to a 30 % difference. This means that the frictional shear stress and, therefore, the surface finish and compressive layer induced by the process will be substantially different. After load increment on the martensitic surface, the friction coefficient increased. Henceforth, it is convenient to take into account the tribological performance of the process (which includes the COF, initial roughness, and initial stress state of the target material [21]) in order to achieve a particular surface integrity depending on the machined microstructure.

### 3.2. Surface Integrity

#### 3.2.1. Surface Roughness

**Figure 5:**
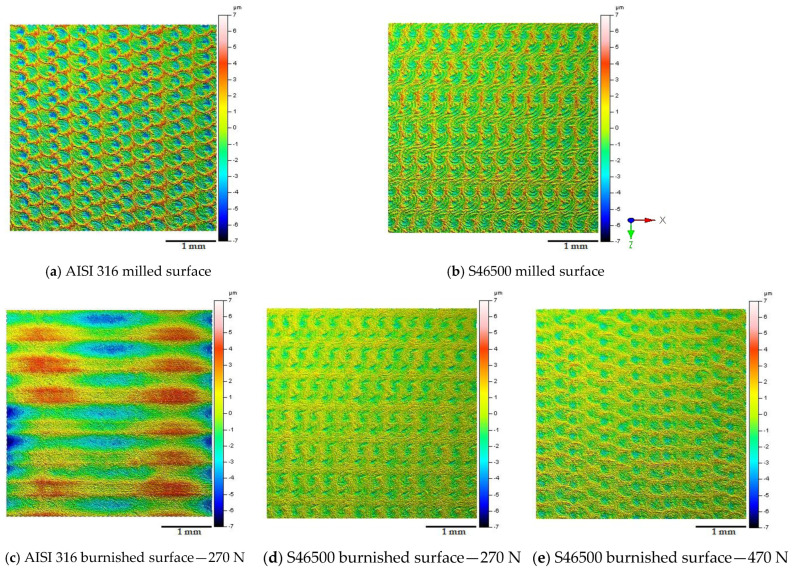
Textured surfaces after milling and burnishing processes at different contact pressures.

Figure 5 displays the milled and burnished areas (4 mm × 4 mm) of each target surface in order to provide the qualitative effect of the burnishing operation under the stated operational and tribological conditions.

**Figure 6:**
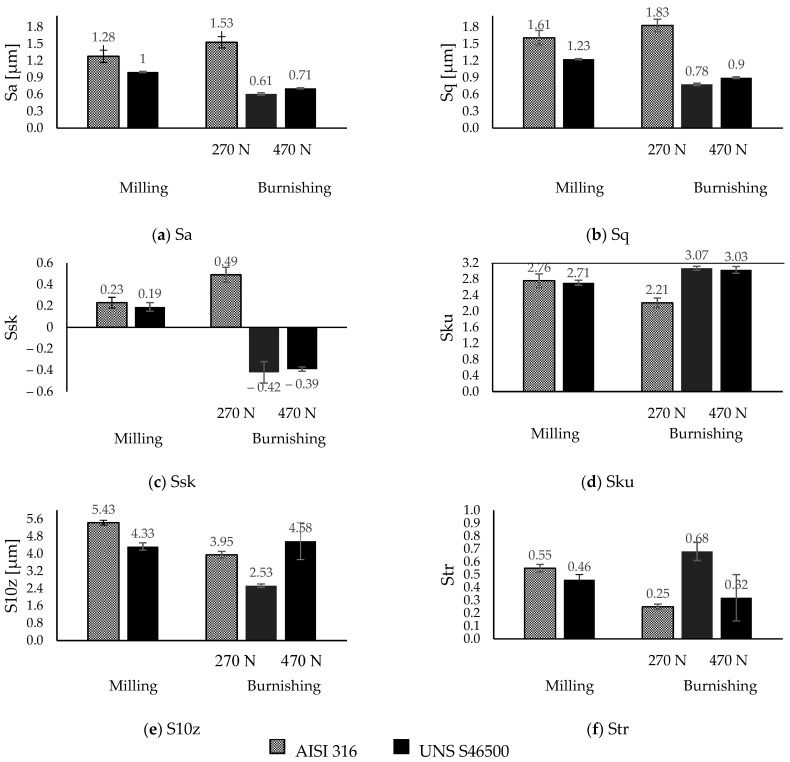
Textured surfaces after milling and burnishing processes at different contact pressures.

It can be observed that the macro-texture conferred by surface milling was more prominent on the austenitic stainless steel than on the martensitic stainless steel. However, under the same burnishing conditions, the texture of the austenitic milled surface disappeared and was replaced by an imprint of the burnishing tool (Figure 5c), showing the extent of the contact area, as well as the magnification of the interaction between the ball and the surface. Regarding the surface finish obtained on the martensitic steel, a softened texture can be seen in comparison with the AISI 316 surface under the same conditions (270 N). It can also be observed that the peaks intensified as the load increased (470 N). Figure 6 summarizes the macro-texture parameters obtained after milling and ball burnishing in order to provide a quantitative description of the analyzed surface modifications.

Based on the height parameters Sa and Sq, a normal distribution of heights (Sa = 0.8 Sq) was evidenced under milling conditions for both materials. This observation was corroborated by the skewness values (Ssk ~ 0) under the same milling conditions. This tendency varied after ball burnishing. Thus, under the same load conditions, the statistical asymmetry after burnishing on AISI 316 stainless steel showed a mass distribution skewed to below the mean plane (Ssk > 0), while the surface of UNS S46500 stainless steel is skewed to above the main plane (Ssk < 0) in equal proportion. The load increment on the second one slightly varied under this condition. With regard to kurtosis (Sku ~ 3), it was observed that the austenitic surface became a non-abrupt platykurtic condition, whereas the martensitic surface responded with a better fit to a Gaussian distribution despite the load increase.

The S10z parameter provides a practical criterion for the statistical behavior of the height. It reveals that the five-point peak height and five-point pit height were 20% more prominent on the austenitic surface. However, this was reduced by 36% on martensitic stainless steel and only 20% on austenitic stainless steel after ball burnishing. This was not consistent with Figure 5c. Since the austenitic crystal lattice had a higher deformation capability (reflected in the COF value), a redistribution of the surface texture was evidenced. Thus, the new average roughness profile may have been displaced below the level of the initial valleys, leading to a loss of tolerance. On the other hand, increasing the pressure on the martensitic surface led to the generation of a pile-up and consequently an increase in the S10z parameter (~15%). This elucidates the marked differences in the surface roughness depending on the stainless-steel microstructure during the ball-burnishing process.

The directional properties quantified through the Str parameter were shown to be moderately isotropic (Str ~ 0.5) after milling for both surfaces. After ball burnishing, the austenitic surface became directionally anisotropic (Str < 0.3), whereas under the same conditions, the martensitic surface increased its isotropy. At a 470 N load, the martensitic surface became anisotropic.

#### 3.2.2. X-ray Difracction

**Figure 7:**
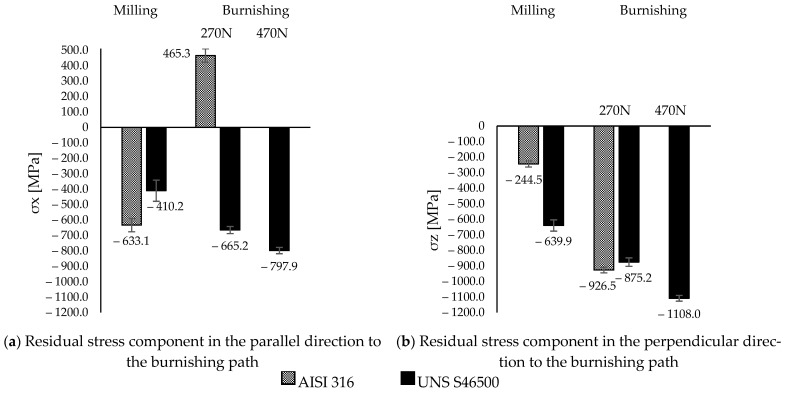
Residual tensor after milling and burnishing processes on stainless steel surfaces.

Figure 7 summarizes the parallel (σx) and perpendicular (σz) tensor components of the burnishing path obtained after the milling and burnishing processes.

The residual tensor component introduced by milling on the austenitic surface was 61% higher in the parallel direction (σx) than in the perpendicular direction (σz) to the burnishing path. The opposite occurred on martensitic and ferritic surfaces [21], where the perpendicular component exceeded the parallel component by 45%. In this manner, although the feed of the milling cutter on the austenitic surface increased the tensor component in its perpendicular direction, on the martensitic surfaces, it increased the tensor parallel to the milling route. However, after burnishing, on the austenitic surface there was a substantial increase in the lower compressive state component (σz) (4 times greater), whereas in the other direction (x-axis), a tensile state was induced. This extended the hypothesis of an anisotropic state independent of the initial tensor on ferritic surfaces [21] to austenitic surfaces. When burnishing was performed on the martensitic surface, the initial anisotropy was reduced by 9% at a 270 N load, whereas with increasing load, the anisotropy increased by 30%. Therefore, the residual isotropy was qualitatively in agreement with the directional isotropy (Str) for both materials after the burnishing process (Section 3.2.1). Regarding the skewness (Ssk), there was no evidence of a directly proportional relationship with the surface tensor, as mentioned in another study [19]. Depending on the microstructure, the surface integrity varied considerably under the same burnishing conditions.

## 4. Discussion

The results elucidate the microstructural impact of the tribological behavior between the burnishing ball and the steel surface. According to Kuznetsov et al. [12], friction constitutes a fundamental parameter to obtain significant improvements or unwanted effects on surface integrity. Therefore, the consequences of high friction involve the generation of uniaxial tensile stress in the rear zone behind the indenter [12], as well as the increase in the pile-up [21]. However, based on the tribo-contact effects on the selected microstructures, the definitions of high and low friction are ambiguous. The allowable tribo-interaction ranges within the process will be given by the limited plastic deformation of the microstructure. In fact, the stress state conditions after ball burnishing with a 270 N load on the austenitic steel show the presence of a tensile state in the burnishing path direction (x-axis), which is in agreement with the approach of Kuznetsov et al. [12]. A clearly detrimental austenitic surface (reflected in the increase in the macro-texture parameters; Figure 6) and a residual anisotropic state [21,22] beyond the compressive condition (Figure 7) were evidenced. As a consequence, a new finish distribution (skipping tolerances) with valleys and peaks defined by the ball track (Figure 5c) was displayed. This high plastic deformation capacity of the austenitic crystallographic lattice allowed for low surface integrity. Nevertheless, under the same conditions (270 N), the textured martensitic stainless-steel surface offered a contrasting microstructural response to the ball-burnishing process. The lower COF (Figure 4) as an effect of the martensitic matrix, determined the displacement of the peaks toward the milling valleys, conferring uniformity on the surface (Figure 5d), whereas the compressive surface state improved in both directions according to the initial trend established by the milling finish. A higher COF (0.17 after load increment) led to the compressive layer’s relocation to the surface (as stated by Kuznetsov et al. [12]), a heightening anisotropy (Figure 7), and an onset of pile-up (Figure 5e, Figure 6e). As seen in Figure 7, the new pressure exerted on the martensitic surface was far from inducing a tensile state in the parallel direction on the burnishing path (which defines high friction [12]) so the hypothesis of high tribo-interactions on this material was limited to the pile-up initiation. Therefore, defining the burnished component’s functionality is pertinent. The process configuration must be prioritized, either the contact interactions with other components (roughness) [3] or the exposure to the corrosion, wear, and fatigue conditions whose resistance improves through the generation or increment of the surface compressive residual state [6,7,8,10,11]. It should be noted that determining the compressive layer’s thickness (sub-surface residual state) as a function of the tribo-interaction degree may modify the high-friction hypothesis of martensitic steels established in this study. Nevertheless, excessive tribo-interaction (overloading) within the process must be prevented in order to allow the treated microstructures to retain some degree of ductility, as cited by Kuznetsov et al. [12].

## 5. Conclusions

In the quest to enhance the surface finish and mechanical properties of stainless steels using a ball-burnishing process, the tribological performance of the process must be considered first. The interaction capability between the ball and the machined surface, quantified by the friction coefficient, defines the surface integrity improvements of the burnished components. Neither the friction value nor its effects are trivial during the ball-burnishing setup. Therefore, to obtain a balance between roughness reduction, design tolerance, and directional and residual anisotropy, or to prioritize one of them, a pertinent COF must be defined. Hence, the present work provides the starting point for a new methodology to parameterize the process based on its interaction (tribological characteristics) at the time when the friction coefficient is delivered as a factual variable in the current numerical methodologies. In addition, this study provides the friction effect on two previously textured microstructures (austenitic and martensitic surfaces) in order to contribute to the guidelines for future ball-burnishing crystalline plasticity numerical conceptualization while demonstrating the process’s capability of enhancing the surface integrity of martensitic stainless steel UNS S46500. The microstructural deformation mechanisms at the local level due to the different tribo-contacts (dislocations, crystallographic orientations, recrystallization, hardness modification, self-hardening), as well as their effect on the thickness of the compressive layer, should be addressed in further research.

## Figures and Tables

**Table 1:** Chemical composition of analyzed steels (in wt %).

Material	Fe	C	Mn	Ti	Cr	Ni	Mo
AISI 316	68.50 ± 0.03	0.02 ± 2 × 10^−4^	1.25 ± 4 × 10^−3^	0.01 ± 1 × 10^−4^	16.69 ± 0.01	9.92 ± 0.02	2.21 ± 0.005
UNS S46500	74.40 ± 0.02	0.01 ± 6 × 10^−4^	0.03 ± 3 × 10^−4^	1.70 ± 0.02	11.69 ± 0.03	10.89 ± 0.01	1.01 ± 6 × 10^−3^

**Table 2:** Initial Milling Conditions.

Tool	Ball Mill UT Coating ø 10 [mm]—Two Teeth
Lateral pass width	0.30 [mm]
Depth of cut	0.20 [mm]
Feed rate	600 [mm/min]
Cutting speed	2000 [rpm]

**Table 3:** Ball-burnishing operational parameters.

Load	270 [N]/470 [N]
Feed rate	600 [mm/min]
Lateral pass width	0.30 [mm]
Vibration-assistance	No

**Table 4:** Mechanical properties of the AISI 316 and UNS S46500 stainless steels.

Material	E [GPa]	ν	σ_0.2_ [MPa]	UTS [MPa]	n
AISI 316	203.6 ± 0.4	0.287 ± 6 × 10^−4^	327 ± 2	588 ± 1	0.307 ± 8 × 10^−4^
UNS S46500	198.8 ± 0.4	0.294 ± 2 × 10^−4^	1571 ± 8	1656 ± 5	0.029 ± 0.002

## Data Availability

The raw/processed data required to reproduce these findings cannot be shared at this time as they also form part of an ongoing study.

## Abbreviations

Ssk	Skewness
XRD	X-ray diffraction
Sa	Arithmetical mean height
Sq	Root mean square height
Sku	Kurtosis
Str	Texture aspect ratio
S10z	Ten-point height
Lc	Parallel length
E	Young’s modulus
ν	Poisson coefficient
σ_0.2_	Yield strength
UTS	Ultimate tensile strength
n	Self-hardening coefficient
*p*_0_	Pressure in the center of the contact region
F	Normal force
R	Indenter radius
E*	Reduced elastic modulus
ASM	American Society of Materials
COF	Coefficient of friction
